# γδ T Cells in Brain Homeostasis and Diseases

**DOI:** 10.3389/fimmu.2022.886397

**Published:** 2022-05-26

**Authors:** Jang Hyun Park, In Kang, Heung Kyu Lee

**Affiliations:** Graduate School of Medical Science and Engineering, Korea Advanced Institute of Science and Technology (KAIST), Daejeon, South Korea

**Keywords:** γδ T cell, central nervous system, brain, neuroimmunology, brain diseases

## Abstract

γδ T cells are a distinct subset of T cells expressing γδ T cell receptor (TCR) rather than αβTCR. Since their discovery, the critical roles of γδ T cells in multiple physiological systems and diseases have been investigated. γδ T cells are preferentially located at mucosal surfaces, such as the gut, although a small subset of γδ T cells can circulate the blood. Additionally, a subset of γδ T cells reside in the meninges in the central nervous system. Recent findings suggest γδ T cells in the meninges have critical roles in brain function and homeostasis. In addition, several lines of evidence have shown γδ T cells can infiltrate the brain parenchyma and regulate inflammatory responses in multiple diseases, including neurodegenerative diseases. Although the importance of γδ T cells in the brain is well established, their roles are still incompletely understood due to the complexity of their biology. Because γδ T cells rapidly respond to changes in brain status and regulate disease progression, understanding the role of γδ T cells in the brain will provide critical information that is essential for interpreting neuroimmune modulation. In this review, we summarize the complex role of γδ T cells in the brain and discuss future directions for research.

## Introduction

γδ T cells are a subset of T cells expressing γδ T cell receptor (TCR) rather than αβTCR. γδ T cell was named after discovery of the γ gene in 1984 (1, 2). Initially, γδ T cells were understudied because they constitute a very minor portion of immune cells and are heterogenous. However, recent studies have emphasized the importance of γδ T cells in a number of diseases. Despite some exceptions, γδ T cells are unrestricted to major histocompatibility complex (MHC) and considered innate immune cells (3). In general, the fate of γδ T cells is already programmed from the thymus, and they do not require complex activation mechanisms (3, 4). Therefore, γδ T cells are rapidly recruited and respond to inflammatory cues. Moreover, γδ T cells regulate adaptive immune responses (5), indicating they are an important bridge connecting innate and adaptive immunity.

γδ T cells are found predominantly at mucosal surfaces rather than lymphoid organs (6). Under steady states, they regulate homeostasis and maintain barrier integrity. Upon infection, they are rapidly activated and regulate immune responses. Vγ5^+^ dendritic epidermis T cells [DETCs; Tonegawa nomenclature (7)] reside in the skin, Vγ7^+^ cells reside in the gut and form intraepithelial cells (IELs), and Vγ6^+^ cells are found in the dermis, vagina, and meninges. Vγ4^+^ T cells have also been observed in the dermis and lung. On the other hand, Vγ1^+^ and Vγ4^+^ T cells, which develop after birth, circulate in the blood or lymphatic fluid (6). In humans, Vδ1^+^ cells usually reside in the mucosal area and Vδ2^+^ T cells are circulating cells, although there are tissue-resident Vδ2^+^ T cells and circulating Vδ1^+^ T cells (8, 9). Although γδ T cells are generally similar across species, murine and human γδ T cells have notable differences (10). Due to the complexity and differences between mouse and human γδ T cells, their investigation is very difficult. For example, classification of murine γδ T cells is dependent on γ chains, whereas human γδ T cells are classified by δ chains (8). In addition, homologous cells for murine Vγ5^+^ DETCs have not been detected in humans (11). Therefore, many aspects of γδ T cell biology remain unclear and further studies are urgently needed to understand their role in immune system function.

Although most mucosal barriers are in contact with the outside and exposed to potential pathogens, meninges are sterile because they encounter the inner side of the central nervous system (CNS) (12). Classically, the CNS has been regarded as an immune privileged organ. A study showed allografts in the CNS were not rejected, unlike allografts in the skin (13). Though circulating immune cells are strongly restricted to enter parenchyma, recent studies re-discovered meningeal lymphatics that drain waste, including CNS antigens (14, 15). Interestingly, antigen presentation in the meningeal spaces and CNS-draining lymph nodes occurs actively (16). Thus, our immune system actually surveils the CNS. However, there are many things concerning the role of the immune system in the CNS that remain elusive. Surprisingly, current data have shown that various immune cells reside or circulate in the meninges (17). Meningeal cytokines interact with parenchymal neurons, astrocytes, or microglia, though the exact mechanisms underlying these interacts are incompletely understood. Meninges-parenchyma interactions regulate multiple neurological functions under homeostasis (18). In addition, meningeal lymphatics and immune system rapidly respond to CNS status and regulate pathology of neurodegenerative diseases and neuroinflammation. γδ T cells are among the multiple immune cells that reside in meninges (19). Recent studies showed meningeal γδ T cells regulate memory formation and behaviors *via* cytokine release (19, 20). Furthermore, parenchymal infiltration and the immunological role of γδ T cells in multiple CNS diseases, including experimental autoimmune encephalomyelitis (EAE), CNS tumors, and infections, have been discovered (8, 21). Because γδ T cells serve as a “safeguard” for the mucosal barrier, γδ T cells are expected to have an indispensable role in the meninges. However, the exact mechanisms concerning how γδ T cells act is lacking. To help identify directions for future studies, we discuss the role of γδ T cells in homeostasis and disease, with a specific focus on the brain.

## γδ T Cells

**Figure 1:**
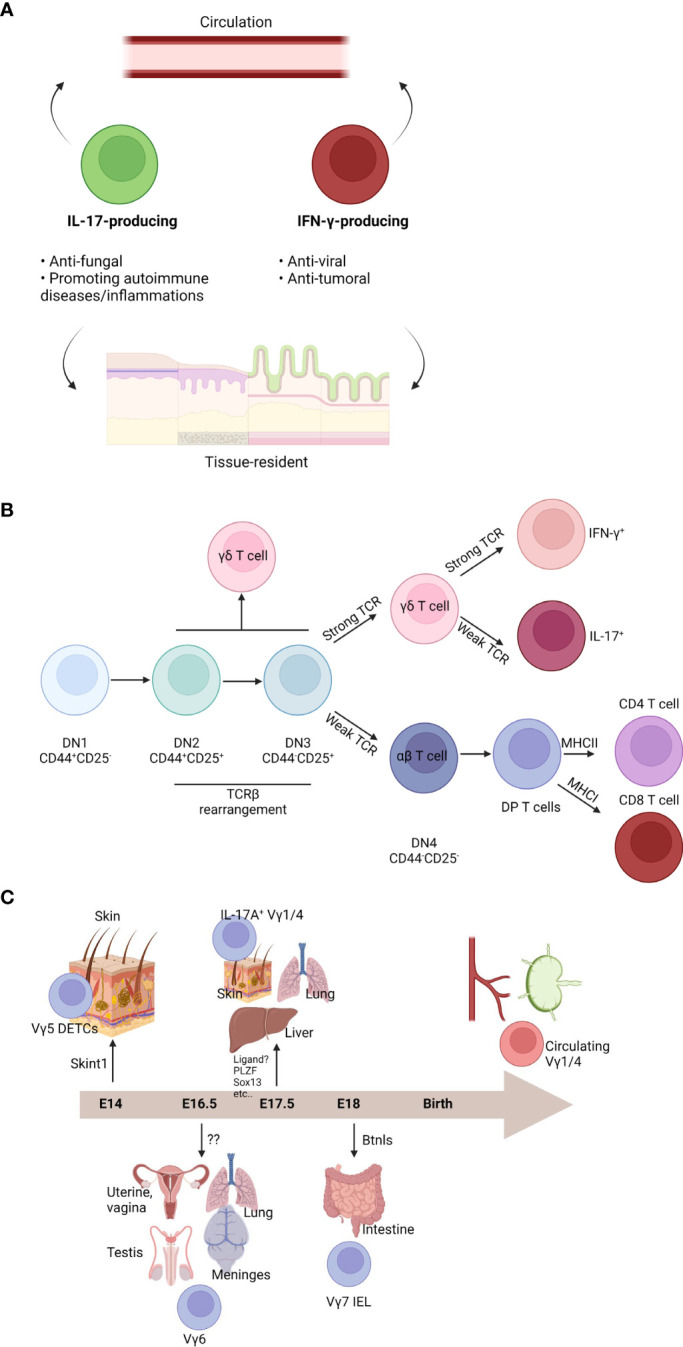
Characteristics and development of γδ T cells. **(A)** γδ T cell subsets are heterogenous. Functionally, γδ T cells can be divided into two groups: one is IL-17-producing cells and the other is IFN-γ-producing cells. IL-17-producing cells are commonly antifungal cells or promoting autoimmune diseases and inflammation. IFN-γ-producing cells are usually antiviral or anti-tumoral cells. Both subsets can be further divided by circulation ability. Although the majority of γδ T cells are tissue-resident cells in the mucosal barriers, some γδ T cells can circulate body. **(B)** T cell development occurs in the thymus. T cell development can be divided by expression of CD44 and CD25 (DN1: CD44+CD25-; DN2: CD44+CD25+; DN3: CD44-CD25+; DN4: CD44-CD25-). Although DN2 or DN3 cells can be γδ T cells, commitment usually occurs after DN3 stage. Strong TCR signal enhances γδ T cell fate. DN4 αβ T cells become CD4+CD8+ DP cells. By their interaction with MHC class I or MHC class II, DP cells become a CD8 T cells or CD4 T cells, respectively. γδ T cells can be IFN-γ-producing cells by strong TCR signal. On the other hand, weak TCR signaling induces IL-17-producing cells. **(C)** Different γδ T cell subsets can be generated in the fetal thymus. At embryonic (E) 14, Vγ5+ dendritic epidermal T cells (DETCs) are generated and migrate into the skin epidermis. SKINT1 is important for Vγ5+ DETC development and selection. At E16.5, Vγ6+ cells can be developed. These cells migrate into the multiple organs such as uterine, vagina, testis, lung, or meninges. They become a tissue-resident cells in those tissues. Cognate ligand for their TCR is not identified. At E17.5, IL-17-producing Vγ1/4+ cells are made. They can migrate into the skin dermis, lung, or liver. They are also tissue-resident cells. Although several factors have been known for their development, cognate TCR ligand is not identified. After E18, Vγ7+ intraepithelial cells (IELs) are generated. They migrate into the gut and become gut-resident cells. BTNL proteins are critical for development and maintenance of Vγ7+ IELs. After birth, Vγ1/4+ cells are further generated. They can circulate and are observed in the blood or lymphoid organs.

T cells are adaptive immune cells that are restricted to MHC-mediated antigen presentation. T cells typically exit from the thymus as naïve cells. Antigen presentation accompanied with multiple inflammatory cues activates T cells and trigger immune reactions (22). However, there are innate-like T cells that have invariant TCRs, such as γδ T cells, natural killer (NK) T cells, and mucosal associated invariant (MAI) T cells (23). γδ T cells are known to be usually independent on MHC-mediated antigen presentation and recognize stress-related molecules, microbial molecules, or phosphoantigens through γδTCR and/or NK receptors, such as NK group 2D (NKG2D) (24). γδ T cells are highly heterogenous and various subsets have been identified. Though some γδTCR ligands have been identified, a comprehensive identification of all ligands is lacking. Functional similarities are shared among multiple γδ T cell subsets and there are two functional subsets. The first functional subset is interferon (IFN)-γ-producing and T helper (Th) 1-like subset and the second functional subset is interleukin (IL)-17-producing and Th17-like subset (**Figure 1A**). The expected roles of γδ T cells are similar to CD4 T cells. IFN-γ-producing γδ T cells are usually antiviral and antitumoral cells, whereas IL-17-producing γδ T cells are antifungal or related to autoimmune diseases such as EAE (8). The detailed functions of γδ T cell subsets are more classified by their circulating capacity. In general, γδ T cells are tissue-resident cells in the mucosal tissues, Vγ5^+^ cells are DETCs in the skin, Vγ4^+^ cells are dermis- or lung-resident cells, Vγ6^+^ cells are residing in vagina, meninges, and dermis, and Vγ7^+^ cells are gut-resident IELs. On the other hand, Vγ4^+^ and Vγ1^+^ cells generated postnatally are circulating cells (6). In humans, Vγ9Vδ2 T cells are predominant circulating γδ T cells, whereas Vδ1^+^ cells and fetal γδ T cells are commonly tissue-resident cells (8, 9). γδ T cells are usually rapidly reacting innate cells that connect innate immune responses to adaptive immune cells and function as a “safeguard”. In addition to their ability to release cytokines, subsets of γδ cells possess NK-like cytotoxicity *via* NK receptors, such as NKG2D (25). However, studying γδ T cells has been technically difficult because of the low number and heterogeneity. Following the recent development of high-throughput analytic tools, such as single cell RNA sequencing, γδ T cell study has progressed tremendously. A number of recent studies have demonstrated the indispensable role of γδ T cells in multiple contexts. Recently, meningeal γδ T cells were identified as a main source of IL-17A in the CNS under homeostasis (19, 20). Currently, cytokines are regarded as neuromodulators because of their ability to directly interact with neurons (18). In addition, IL-17A is one of the most important cytokines for the neurological system and Vγ6^+^ cells, which reside in meninges, produce IL-17A (19). On the other hand, other γδ T cells can invade into the parenchyma under disease conditions and regulate multiple immune responses. For example, circulating γδ T cells can invade into glioblastoma multiforme (GBM) tissues, leading to antitumor responses (26). Although γδ T cells seem to be critical immune cells in the CNS, many aspects of their biology remained unclear.

## Development and Maintenance of γδ T Cells

### γδ T Cell Development

Similar to other T cells, γδ T cells are generated from the thymus (23). Common lymphoid progenitor cells from the bone marrow enter the thymus and become CD4^-^CD8^-^ double negative (DN) T cells. DN T cells are subdivided into four differentiation stages (DN1: CD44^+^CD25^-^; DN2: CD44^+^CD25^+^; DN3: CD44^-^CD25^+^; DN4: CD44^-^CD25^-^) (**Figure 1B**). During the DN stage, pre-TCR are formed when pre-TCRα and TCRβ rearrangement induces progression into the CD4^+^CD8^+^ double positive (DP) stage. Then, DP T cells interact with cortical epithelial cells expressing MHC molecules with self-antigens, which leads to a selection process where too weak signaling induces DP cell apoptosis. Moderately reactive DP T cells become single positive (SP) T cells. Thymocytes that interact with MHC class I become CD8 T cells and cells what interact with MHC class II become CD4 T cells or initial signaling strength determines fates of T cells (27, 28). SP T cells are further selected by negative selection by medullary epithelial cells. Other unconventional T cells, such as NKT cells and MAIT cells, are generated from the DP stages. Uniquely, γδ T cells develop from the DN stages (23). γδ T cell fate is commonly determined at the DN3 stage. However, some γδ T cell subsets are derived from the DN1 or DN2 stages. In mice, γδ T cell development begins in the fetal thymus and γδ T cells constitute the major T cell subset at this early stage due to a lack of αβ T cell development (29). Initial mouse γδ T cell development occurs in the fetal thymus, generating DETCs expressing Vγ5 (**Figure 1C**). At embryonic (E) 14, DETCs are produced and preferentially migrate into the epidermis (30). Interestingly, a study revealed DETCs do not originate from hematopoiesis in bone marrow. However, DETC progenitors were derived from yolk sac like Langerhans cells (31). Vγ6^+^ cells are a type of intraepithelial lymphocytes (IELs) of reproductive organs and meninges. Vγ6^+^ cells usually express IL-17A and develop at E16.5. Vγ4^+^ and Vγ1^+^ IL-17A-producing cells develop at E17.5 (32). Development of gut-homing Vγ7^+^ IELs begins at E18 and continues postnatally (30). Some intestinal IELs are thought to be developed extrathymically (33). Some IFN-γ-producing liver-resident γδ T cells are extrathymically developed from Lin^-^Sca-1^+^Mac1^+^ hematopoietic stem cells and progenitor cells in the liver (34). Similarly, human γδ T cells arise from the fetal liver (35). Vγ9Vδ2 T cells can be observed at the fetal liver at 5-7 weeks gestation, whereas thymic Vγ9Vδ2 T cells are detected at 8 weeks gestation (36). Fetal Vγ9Vδ2 T cells are relatively invariant and have public clones. Postnatally, Vγ9Vδ2 T cells are rarely generated, whereas Vδ1^+^ and Vδ3^+^ T cells are preferentially generated. TCR repertoire of Vδ1^+^ and Vδ3^+^ T cells is largely dependent on microbial exposure (37). Although fetal Vγ9Vδ2 T cells slowly turn over and have self-renewal capacity, adult-derived Vγ9Vδ2 T cells can also be generated and be a major source human γδ T cells in the blood (38). Recent observation showed the fetal thymus produces hybrid T cells that expressing both αβTCR and γδTCR (39). These hybrid cells, which can produce IFN-γ, IL-17A, and granulocyte-macrophage colony-stimulating factor, are hyperactive. The hybrid cells underwent positive αβ-selection.

After birth, the majority of newly generated γδ T cells are Vγ4^+^ and Vγ1^+^ cells. Although both cells can produce IL-17 and/or IFN-γ, Vγ1^+^ cells are usually association with IFN-γ production and Vγ4^+^ cells are commonly associated with IL-17A production (6). Their fate is determined during thymic development. CD27^+^CD44^int^ cells actively secrete IFN-γ, whereas CD27^-^CD44^hi^ cells produce IL-17A (8). As this process is not well understood, identifying factors that determine γδ T cell fate has been of great interest. Although various factors can be involved, TCR strength may be the most important factor for determining γδ T cell fate. Before γδ T cell commitment, TCR strength is important for γδ T cell identity. If γδTCR is weak, cells tend to preferentially differentiate into αβ T cells (40). These commitments are known to occur after TCR expression. It was dependent on extracellular signal-regulated kinases (ERKs)-mediated early growth response activation (41). Overexpression of friend leukemia integration 1 (Fli1) prevents progression of DN T cells into DP T cells (42). As a result, Fli1 overexpression may create a preferential environment for γδ T cell development, which was mediated by strong TCR mimicry. Strong TCR activation results in CD73 expression. Although CD73^-^ γδ T cells retain the potential develop into αβ T cells, CD73^+^ cells commonly become γδ T cells (43). After γδ T cell commitment, TCR strength may determine whether the γδ T cells become IL-17-producers or IFN-γ-producers. Usually, a strong TCR signal tends to make γδ T cells become a CD44^+^CD45RB^+^T-bet^+^ IFN-γ-producing cells. On the other hand, a weak TCR signal induces CD44^hi^RORγt^+^ IL-17A-producing γδ T cells (44). This mechanism was dependent on the ERK pathway. Mechanistic target of rapamycin (mTOR) complex 1 (mTORC1) and Notch signaling also determine αβ/γδ fate *via* metabolism (45). Likewise, metabolic pathways are also important for γδ T cell fate. IFN-γ-producing cells are dependent on glycolysis and IL-17A-producing cells are dependent on oxidative phosphorylation. These dependencies are imprinted from thymic development to peripheral maintenance (46). Environmental cytokines also regulate the function of γδ T cells. For example, IL-1β and IL-23 induce extrathymic commitment of CD27^+^CD122^-^ Vγ4^+^ cells to become an IL-17A-producer (47). Vγ4^+^ T cells that have never made IL-17A can produce IL-17A *de novo* by IL-1β and IL-23 (48). In parallel, IFN-γ-producing cells can be generated by IL-12 and IL-18 (49). Transcription factors are also important regulators of γδ T cell fate. Fetal-derived γδ T cells may be marked by promyelocytic leukemia zinc finger protein (PLZF) (50, 51). IFN-γ^+^ γδ T cells need T-bet, but not Eomes. On the other hand, IL-17A^+^ γδ T cells need RORγt, but not RORα and BATF (52). Co-stimulatory molecules, such as CD27 or ICOS, also support γδ T cell fate determination (53, 54).

### Ligands for γδTCR

As mentioned above, TCR signaling is important for γδ T cell development and maintenance. Thus, identifying γδTCR ligands and their roles is indispensable to further understand γδ T cell biology. Though major subsets of γδ T cells are not dependent on MHC-mediated antigen presentation, γδ T cells are dependent on MHC-like molecules, stress-induced molecules, and phosphoantigens (24). The most well-known γδTCR ligands are selection and upkeep of intraepithelial T cells protein 1 (SKINT1) and butyrophilin-like proteins (BTNL) molecules (**Figure 1C**). Vγ5^+^ DETCs are dependent on SKINT1 (55). SKINT1 expression is restricted to the thymus and skin keratinocytes. SKINT1-mediated TCR signaling is not only important for development of DETCs, but also epidermal maintenance (56). Likewise, BTNL molecules are important for Vγ7^+^ IELs. BTNL1 and BTNL6 are necessary for murine Vγ7^+^ IELs and BTNL3 and BTNL8 are needed for human intestinal Vγ4^+^ T cells (57). T10/22, a MHC class Ib molecule, is also important for γδ T cell development (58). The most well-known γδTCR ligands in humans are BTN3A1 and BTN2A1. Phosphoantigens induce a conformational change in BTN3A1-BTN2A1 dimers, which binds to Vγ9Vδ2 TCR (59). Endothelial protein C receptor (EPCR)-Vγ4Vδ5 TCR (60), Annexin A2-Vδ2 TCR (61), tRNA synthetases-Vγ3Vδ2 TCR (62), ephrin type-A receptor 2 (EphA2)-Vγ9Vδ1 TCR (63), and R-phycoerythrin-Vδ1 TCR (64), CD1c/d-Vδ1 TCR have been reported (65, 66). Contrary to a number of reports that argued fetal thymus-derived γδ T cells are invariant, adult-derived γδ T cells have relatively variant TCR chains (67). Likewise, there are some γδ T cell subsets that are dependent on MHC-mediated antigen presentation (68). Thus, studying γδ T cells and their ligands is complex. In some cases, γδ T cells can be activated without TCR signaling, but activated by stress-induced molecules, such as MHC class I chain-related protein A/B (MICA/B) or retinoic acid early inducible 1 (Rae-1), *via* NKG2D receptor (8, 69). In conclusion, TCR ligands should be considered in the context-dependent manner to understand the role of γδTCR. A study showed murine γδTCR depletion antibodies could not remove γδ T cells, but made the cells undetectable *via* intracellular uptake of γδTCR (70). Because this system depletes functional γδTCR from cellular surfaces, γδTCR depletion antibodies could be used to investigating the role of γδTCR. Unfortunately, ligands for Vγ6^+^ cells have not been identified. However, administration of anti-γδTCR inhibits meningeal γδ T cell functions (19). Thus, TCR-mediated signal is required for cytokine secretion in the meninges. Identifying the ligand(s) that regulate meningeal γδ T cell homeostasis and activation is critical to understand the role of γδ T cells in brain physiology.

## γδ T Cells in Brain Homeostasis

### Maintenance and Recruitment of Brain γδ T Cells

Vγ6^+^ cells, which are enriched in the meninges, reproductive organs, and dermis, are the major γδ T cell subset in these organs (6). In addition, they are a major source of IL-17A; however, they do not express IFN-γ. Although a study claimed ZAP70-deficient mice had less IL-17A-producing γδ T cells, including Vγ6^+^ cells, compared to wild type (WT) mice (71), previous study has proposed that weak TCR signaling is important for development of IL-17A-producing γδ T cells, including Vγ6^+^ cells (44). It is important to note that the dispensable role of TCR signaling in thymic development of γδ T cells does not mean that it is also dispensable for peripheral maintenance and cytokine secretion. A series of studies have emphasized that tonic TCR signal from tissue-specific niches is important for maintaining tissue-resident γδ T cells (72, 73). Vγ6^+^ cells γδ T cells being developing at E.17.5 (23). Furthermore, experiments using bone marrow chimeras demonstrated that adult thymus could not produce IL-17A-producing γδ T cells, suggesting Vγ6^+^ cells may be fetal-derived, self-renewing, and long-lived cells (32). However, it remains unclear how Vγ6^+^ cells are recruited into the meninges and maintained. In the uterus, Vγ6^+^ cells are the dominant γδ T cells in homeostasis (74). However, pregnancy induces recruitment of Vγ4^+^ cells into the placenta (75). Although the relation of Vγ4^+^, Vγ6^+^ cells, or IL-17A to outcomes of pregnancy is controversial, allogenic pregnancy experiments revealed that recruitment of γδ T cells in the uterus is dependent on allotype (75, 76). In parallel, certain inflammatory cues can recruit different γδ T cell subsets in the meninges or brain parenchyma (21, 77). It has shown that brain injury or inflammation can recruit Vγ1^+^, 4^+^, 6^+^ cells in the parenchyma (78–80). CCR6 is important for migration of IL-17-producing γδ T cells (81), and a study showed most meningeal γδ T cells expressed CCR6 (20). However, another study showed meningeal γδ T cells expressed large amounts of *Cxcr6* and *Ccr2.* In addition, *Cxcr6*-deficient mice showed γδ T cell reduction in the meninges (19) and their functions may be dependent on γδTCR, but not cytokines, such as IL-1β or IL-23 under homeostasis (19, 20). However, other factors affecting meningeal γδ T cells should be further addressed. Taken together, meningeal γδ T cells have crucial roles maintaining brain homeostasis and behaviors of animals. However, further study is needed to uncover the exact mechanisms governing how they are recruited, activated, and maintained.

### The Role of Meningeal γδ T Cells in the Homeostatic Brain

**Figure 2:**
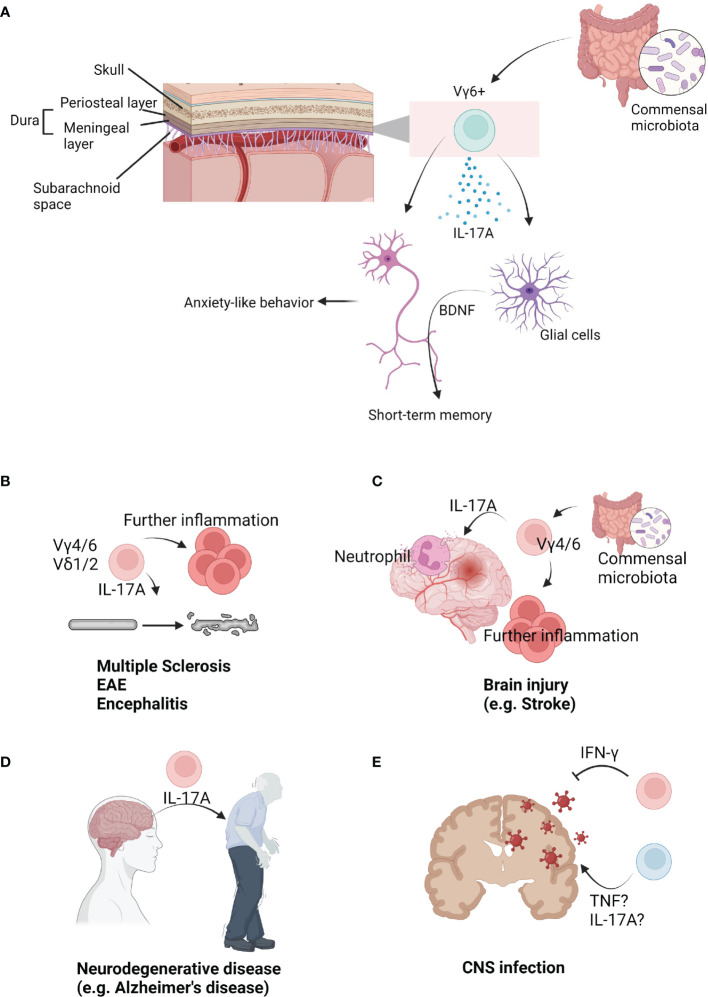
The role of γδ T cells in brain immunology. **(A)** Upon brain parenchyma, multiple layers surround brain. Under skull, dura mater (periosteal layer, meningeal layer) is situated. Under meningeal layer, arachnoid and subarachnoid space exist. In the meninges, Vγ6+ cells are populated. They seem to be affected by commensal microbiota. Under steady state, Vγ6+ cells produce IL-17A. IL-17A from meninges can be delivered into the parenchyma. Direct signal from IL-17A into neurons can regulate anxiety-like behavior. On the other hand, IL-17A can regulate short-term memory via glial BDNF. **(B)** γδ T cells are related to progression and severity of brain autoimmune diseases. Mouse Vγ4/6+ cells or human Vδ1/2+ cells are known to be related to these diseases. Usually, IL-17A from γδ T cells initiate or further promote diseases. **(C)** γδ T cells are involved in injury-induced inflammation in the brain. Vγ4/6+ cells usually produce IL-17A which recruits neutrophils. They are known to be regulated by commensal microbiota. As an early inducer, γδ T cells further promote inflammations. **(D)** γδ T cells are also related to neurodegenerative diseases. IL-17A may be strongly associated with development of diseases such as Alzheimer’s disease. **(E)** γδ T cells can infiltrate into the infected brains. Multiple pathogens can infect into the brain. Usually, IFN-γ-producing γδ T cells resolve viral infections. However, TNF or IL-17A is associated with infection-induced inflammation.

Decades ago, heat shock protein 70 (HSP70) was the most well-known ligand for human multiple sclerosis (MS) γδ T cells (82). Interestingly, a study observed that oligodendrocytes, postischemic neurons, and microglia express HSP70 under heat exposure (83). This study suggested γδ T cells may be cytotoxic to brain cells. Also, this study revealed that different types of γδTCRs are expressed in the cortex, hypothalamus, and medulla of postmortem samples. Another study showed that normal CNS tissue contains γδ T cells (84). Although this study may have technical limitations, the γδ T cells from normal CNS tissue expressed low CD45RB levels, which may suggest these cells are meningeal IL-17A-producing cells. Currently, many people agree that γδ T cells do not exist in the normal CNS parenchyma. However, a large amount of γδ T cells are present in the meninges (**Figure 2A**) (19). Furthermore, these cells are IL-17A-producing cells, but not IFN-γ-producing or IL-22-producing. Also, these cells are rarely observed in the arachnoid and choroid plexus. This study also showed that meningeal γδ T cells are present three days after the postnatal period (P3). They showed tissue-resident phenotypes that were not derived from circulation. Adult meningeal γδ T cells were not Ki67^+^ and showed poor incorporation of BrdU, indicating they are not proliferative and self-renewal. They produce IL-17 under steady states, which may be dependent on TCR signaling. Commensal-derived signaling also contributes to γδ T cell IL-17A production. However, the number of meningeal γδ T cells was not dependent on bacterial signals. This study also revealed that meningeal γδ T cell-derived IL-17A regulates anxiety-like behaviors of mice. Although how meninges-derived cytokines arrive at parenchyma is unclear, IL-17A can directly affect excitatory glutamatergic neurons in the medial prefrontal cortex (mPFC). Notably, IL-17 receptor A (IL-17Ra) is expressed by multiple brain regions. A direct IL-17A signal may promote neurotransmitter release from excitatory presynaptic terminals of mPFC neurons to induce anxiety-like behaviors. However, IL-17A did not affect intrinsic neuronal excitation. This finding may explain how animals can rapidly respond to environmental stresses. On the other hand, *Tcrd*-deficiency did not affect spatial memory task performance, social preference, or foraging behavior. According to an interesting study by the Ribot group, *Tcrd*-deficient mice did not show deficits in exploratory behavior, motor function, and anxiety (20). However, these animals showed impaired short-term spatial working memory, but not long-term memory formation. Critically, these findings were dependent on IL-17A. IL-17A directly signals to glial cells inducing production of brain-derived neurotrophic factor (BDNF) in glial culture system. However, because these phenotypes were not repeated under microglia- or astrocyte-specific deletion of IL-17R, direct evidence linking IL-17A and memory formation is still lacking and should be further addressed. Nonetheless, IL-17A-mediated BDNF seems to be involved in long-term potentiation of neurons during short-term memory formation. Taken together, γδ T cells, as main source of IL-17A, regulate multiple functions of the brain under steady states.

Maternal IL-17A is also important for progeny behavior. Poly I:C-induced maternal immune activation (MIA) mimicking infections showed autism-like behavior of progenies (85). Because *Il17a* expression was not detected in fetal brain at E14.5, IL-17A may be derived from the mother under MIA. MIA resulted in impaired cortex development of offspring. Given the authors showed conditional deletion of *Rorc* using CD4-Cre mice, they concluded CD4 T cells are responsible for IL-17A production. This data excluded participation of γδ T cells, lymphoid tissue inducer cells, and innate lymphoid cell type 3s. In addition, intestinal dendritic cells stimulate CD4 T cells *via* IL-1β, IL-23, and IL-6, which leads to IL-17A production in a maternal microbiota-dependent manner (86). Although they clearly showed CD4 T cells are critical, the contribution of uterine γδ T cells or fetal γδ T cells to behavioral impairment in offspring would be an interesting study to explore. Moreover, dietary salt also induces CD4 T cells to produce IL-17A *via* serum/glucocorticoid regulated kinase 1 (SGK1) (87). Similarly, IL-17A-inhibiting *Lactobacillus murinus* was reversed by salt-uptake, resulting in elevated IL-17A (88). Maternal salt uptake also induces abnormal behaviors of offspring (89, 90). Dietary salt has been shown to induce cognitive dysfunction by gut-initiated Th17 responses (91). Taken together, maternal CD4 T cell-derived IL-17A affects offspring cognitive functions and behaviors. In addition, the role of γδ T cells in MIA-induced autism-like behaviors and cognitive dysfunction under salt uptake or other environmental changes should also be addressed. On the other hand, intrauterine inflammation without systemic inflammation induces neutrophil infiltration into the decidua. In parallel, neutrophils and macrophages were increased in the fetal liver. In the fetal brain, granulocytes and activated microglia were increased. Among immune cells, Gr1^+^ γδ T cells were the most rapidly responding cells, which produce IFN-γ rather than IL-17A (92). Thus, other kinds of MIA rather than systemic poly I:C should be also considered.

## The Role of γδ T Cells in Brain Diseases

### Autoimmune Diseases in CNS

In 1991, it was revealed that human peripheral blood-derived γδ T cells can kill fresh human brain-derived oligodendrocytes *ex vivo* (93). Furthermore, γδ T cells were observable in the plaques and cerebrospinal fluid (CSF) of MS patients. This study suggested the possibility of γδ T cell participation in MS progression. Although CD4 T cells are important for chronic MS, γδ T cells were the most activated cells in recent onset MS patients (94), and the activated γδ T cells were oligoclonal. This study suggested γδ T cells can be expanded by MS antigens and are the initiating cells in MS pathology (**Figure 2B**). Demonstrated with a murine EAE model, administration of anti-γδTCR (UC7-13D5) worsened EAE pathology (95). These data suggested the regulatory role of γδ T cells in disease progression. As mentioned above, anti-γδTCR administration does not deplete γδ T cells, rather it inhibits TCR signaling (70). Thus, this finding showed TCR-reactive γδ T cells have regulatory role in the EAE. Another study using a murine EAE model revealed γδ T cells are associated with IFN-γ levels (96). On the other hand, early IL-17A production from γδ T cells promotes later activation of Th17 cells (97), indicating heterogenous γδ T cells participate in MS or EAE. In human samples, Vδ1^+^ cells were largely observed in the blood and CSF of MS patients. On the other hand, Vδ2^+^ T cells have strong cytotoxicity against oligodendrocytes (98). Under MS, long-term treatment of IFN-β expands Vδ1^-^Vδ2^-^Vγ9^-^ γδ T cells, which were related to better outcome of MS patients (99). Taken together, human data also suggested a heterogenous role of γδ T cells in the MS progression. In the murine EAE model, γδ T cells infiltrate into the brain parenchyma using integrin beta 2 family, and its expression was rapidly reduced after infiltration (100). Another study showed that gut *L. acidipiscis* reduces Vγ4^+^ cells while Vγ1^+^ cells were increased. Because gut *L. acidipiscis* was related to better EAE outcomes, Vγ4^+^ and Vγ1^+^ cells may have opposing roles (101). IFN-γ-producing and IL-17A-producing γδ T cells have been shown to have opposing roles as IFN-γ- or IFN-γR-deficient mice have enhanced EAE (102, 103). It would be interesting to investigate the contribution of meninges-derived IL-17A or Vγ6^+^ cells using an EAE murine model. γδ T cells are also related to Rasmussen’s encephalitis (RE) pathology. Although CD8 T cell response is critical for RE inflammation, more innate cell types could be associated with disease initiation (104). This study revealed Vδ1^+^ cell clonal expansion in the parenchyma of RE patients. Because microglial activation *via* TLRs can enhance IL-17A-producing γδ T cells through IL-1 and IL-23, microglial inflammation can be a trigger for multiple CNS inflammations (105).

### γδ T Cells in Brain Injury

Infiltration of γδ T cells in the brain parenchyma is also observable following ischemic injury (106). While CD4 T cells induce tumor necrosis factor (TNF) production by macrophages *via* IFN-γ, γδ T cells promote neutrophil infiltration through IL-17A (**Figure 2C**). IL-17A and TNF synergistically induce CXCL1 expression by astrocytes, which further promotes neutrophil infiltration (107). Another interesting study showed intestinal microbiota regulates outcomes of ischemic stroke *via* γδ T cells. Intestinal microbiota regulates dendritic cells, which promotes γδ T cell activation. IL-17A produced from γδ T cells enhances stroke pathology. On the other hand, antibiotics uptake increases Tregs and reduces γδ T cells resulting in better outcomes for stroke mice (77). Taken together, IL-17A from γδ T cells is a critical cytokine that promotes inflammation after brain injury. Two studies showed IL-17A is predominantly expressed by infiltrating Vγ4^+^ or Vγ6^+^ cells (79, 108), and CCR6 seems to be important for Vγ4^+^ or Vγ6^+^ cell migration. Furthermore, the regulatory role of γδ T cells was demonstrated using a NaIO3-mediated retinal pigment epithelium injury model. γδ T cells produce IL-4 and IL-10 to reduce injury in an aryl hydrocarbon receptor (AhR)-dependent manner (109). In the case of perinatal brain injury, injury delays neurophysiological maturation. This was related to gut microbiota, *Klebsiella*, which has been associated with an increase in γδ T cells expressing IL-17A and VEGF-A (110). On the other hand, both the Kipnis group and Colonna group showed that skull bone marrow provides myeloid cells and B cells to the meninges and parenchyma (111, 112). Direct production of immune cells *via* skull bone marrow might be involved in brain injury progression. However, these two studies suggested T cells are derived from the peripheral blood, not the skull bone marrow. It may be due to T cell maturation occurs at the thymus. However, *de novo* development of γδ T cells in the skull bone marrow or meninges should be experimentally tested to clarify this. Also, γδ T cells promote bone regeneration after injury *via* IL-17 (113). Thus, meningeal γδ T cell-derived IL-17 may be able to regulate skull regeneration resulting in recovery after brain injury.

### Neurodegenerative Diseases

A number of studies have shown that inflammation is associated with severity of neurodegenerative diseases, including dementia, Parkinson’s diseases, and Huntington’s diseases (114). Clonal expansion and antigen reactivity of T cells have been observed in multiple neurodegenerative diseases (115–117). Because microglial-intrinsic inflammatory gene regulation can induce T cell infiltration in the parenchyma and neuroinflammation (118), immune reaction may be associated with initiation and development of multiple neurodegenerative diseases. During the initial stage of MS, pioneer cells enter the CNS and initiate further inflammation without pathologies (119). On the other hand, γδ T cell activation, rather than αβ T cells, has been observed in CNS inflammation in early onset MS (94, 106). Thus, γδ T cells may regulate the first wave of neuroinflammation in neurodegenerative diseases, though there is no direct evidence conclusively demonstrating this. *TRG* genes can be detected in both the human brain and blood. The brain has less *TRGV9* clones than the blood. However, the brain contains more *TRGV2, 4*, and *8* genes. In this study, it was shown that aging is known to reduce the *TRG* repertoire. In addition, an Alzheimer’s disease (AD)-associated *TRG* pattern was observed among AD patients (120). This study has technical limitations because tissues were not perfused and *TRG* transcript could be expressed by non-T cell lineages (121). Nonetheless, these data suggest a possible relationship between γδ T cells and AD. Consistently, IL-17-producing cells, including γδ T cells, accumulate in the brain and meninges of the 3xTg-AD mouse model (122). This study demonstrated IL-17 triggers AD onset independent of amyloid β and tau pathology (**Figure 2D). ** Thus, γδ T cells may be a “pioneer cells” of neurodegenerative diseases. Likewise, γδ T cells were increased in the blood and CSF from Parkinson’s disease (PD) patients compared to other neurological diseases (123). In summary, γδ T cells can contribute to progression and initiation of multiple neurodegenerative diseases. Despite the lack of a direct connection, γδ T cells may be related to early trigger of diseases. The diverse roles and mechanisms of γδ T cells in multiple neurodegenerative diseases should be further addressed.

### Brain Infections

Microbe infections can also induce neuroinflammation and neurological symptoms. For example, toxoplasma infection can induce toxoplasmic encephalitis. A study showed IL-6 deficiency was associated with more cyst and necrosis of the brain. IL-6 knock out mice have more CD8 T cells and less CD4 T cells and γδ T cells compared to WT mice (124). This suggested γδ T cells may be related to inflammation in toxoplasmic encephalitis. Malaria infection can also induce brain inflammation. Infection by *Plasmodium yoelii* induces brain inflammation of BALB/c mice. However, DBA/2 mice are resistant to infection. IL-2-mediated γδ T cell infiltration in the brain was critical for susceptibility to *Plasmodium yoelii* infection (125). Another study also showed γδ T cell deficiency reduced intracranial mesocestoides corti-mediated neurocysticercosis pathology (126). Thus, γδ T cells contribute to infection-induced brain inflammation (**Figure 2E**).

γδ T cell infiltration was observed following West Nile virus (WNV) infection. The majority of infiltrating γδ T cells were Vγ1^+^ and Vγ4^+^ cells that produce IFN-γ and TNF, respectively (127). IFN-γ has antiviral functions, whereas TNF was associated with worse symptoms. This study also showed aging increases Vγ4^+^ cells but reduces Vγ1^+^ cells. Vγ4^+^ cells also produce IL-17A following WNV infection (128). According to this study, Vγ4^+^ cells also inhibited the Vγ1^+^ cell response and associated IL-10 production. Regarding oral herpes simplex virus type 1 (HSV-1) infection, C57BL/6 mice are resistant to infection while BALB/c mice are susceptible. In C57BL/6 mice, HSV-1 replication is limited to the brain stem. However, HSV-1 replication was observed throughout the whole CNS in BALB/c mice. Although CD8 T cells, NK cells, and NKT cells were crucial for limiting viral infection in the CNS, γδ T cells were important for inhibiting viral spreading in the trigeminal ganglia (129). Epstein-Barr virus (EBV) is one of the most important CNS viruses because it is largely related to MS progression and onset. Longitudinal analysis showed that high prevalence of EBV is related to MS (130). Consistently, a study showed antibodies derived from clonally expanded B cells in MS can bind to EBV Epstein-Barr nuclear antigen 1 (EBNA1) and CNS-derived GlialCAM protein. Furthermore, the presence of EBNA1/GlialCAM antibodies was associated with severe MS (131). A study showed EBV reactivation after hematopoietic stem cell transfer was negatively correlated with Vδ2^+^ T cells (132). This study showed γδ T cells exhibit cytotoxicity against EBV-infected cells *in vitro*. Thus, γδ T cells may have role in EBV-mediated MS. Likewise, γδ T cells are highly associated with cytomegalovirus (CMV) infection (133). Because herpesviruses such as human CMV or HSV seem to be related to multiple neurodegenerative diseases (134–136), γδ T cells may have critical role preventing CNS viral infection-mediated neurological disorders.

### Brain Tumors

**Figure 3:**
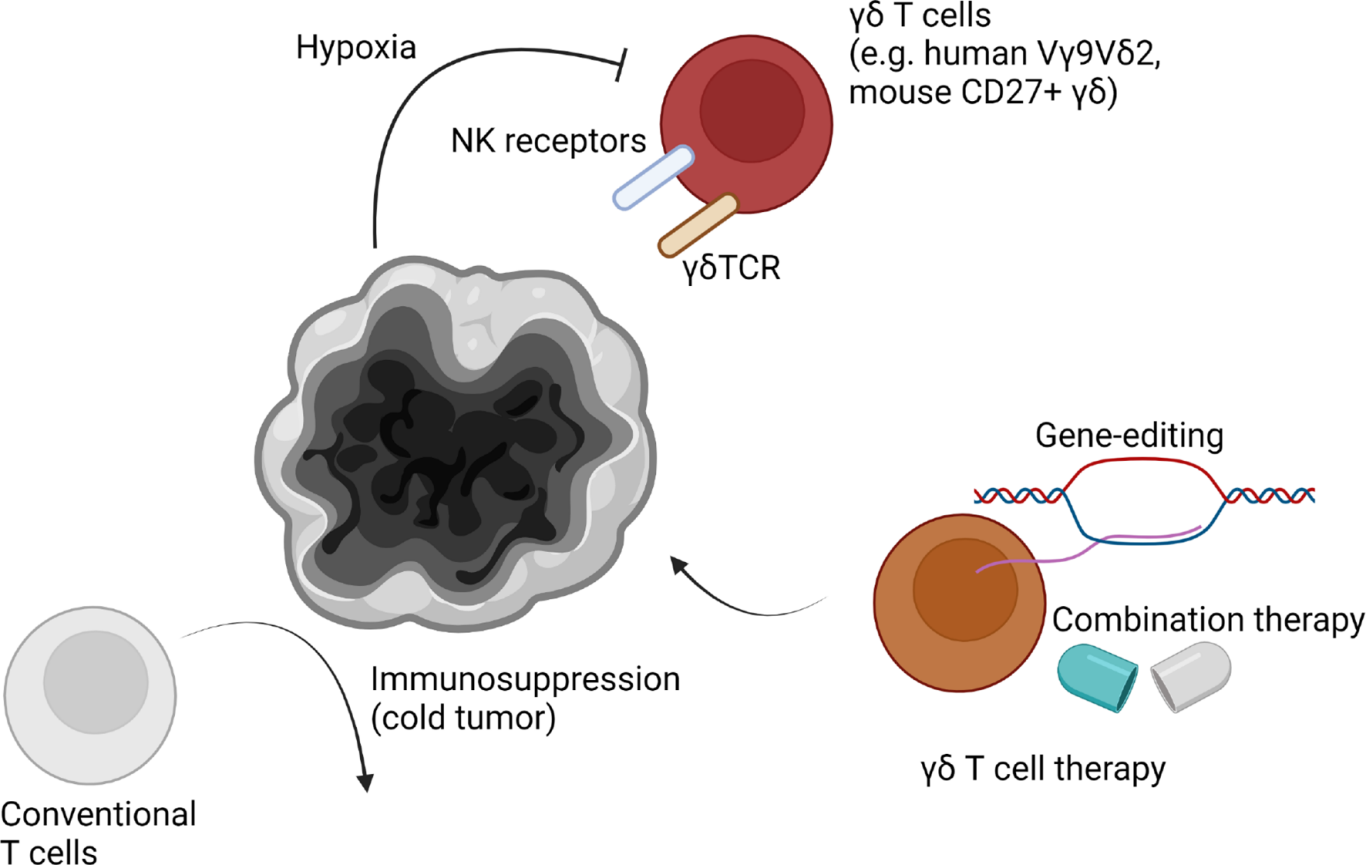
γδ T cells in the brain tumor microenvironment. High-grade brain tumors such as glioblastoma multiforme (GBM) are known to be immunosuppressive “cold tumors”. Due to strong immunosuppression mechanisms, conventional T cells are malfunctional. However, γδ T cells are known to be potent strong anti-brain tumor immune cells. Human Vγ9Vδ2 T cells or murine CD27+ γδ T cells are known to infiltrate the tumor microenvironment. They may fight with tumor cells through both of γδTCR and NK receptors, including NKG2D. However, γδ T cell reactivity is suppressed in the brain tumor microenvironment via severe hypoxia. Additionally, other mechanisms such as chemotherapy-induced cell death can be involved in suppression of γδ T cell reactivity. Thus, gene-editing to generate resistant γδ T cells or developing combination therapies can enhance γδ T cell immunity in the brain tumor microenvironment.

Recently, the role of γδ T cells in multiple tumors has been emphasized. A study showed γδ T cell were mostly correlated to better prognosis among multiple tumor-infiltrating immune cells (137). Different subsets of γδ T cells can be identified in the tumor microenvironment (**Figure 3**). Functionally, γδ T cells can be subdivided into IL-17A-producing cells and IFN-γ-producing cells (8). IFN-γ-producing cells tend to be cytotoxic cells, with some exceptions. A recent study showed IL-17A-producing γδ T cells are protumor cells and IFN-γ-producing cells are antitumor cells using subcutaneous murine tumor models (46). This tendency was conserved across multiple tumors (8). Also, our group showed γδ T cells are associated with longer survival of brain tumor patients (138). However, αβ T cells showed the opposite tendency. Meanwhile, using a murine high-grade glioma (HGG) model, we showed depletion of NK cells, γδ T cells, CD8 T cells, or CD4 T cells did not affect survival of HGG-bearing mice. We discovered that hypoxia was positively related to increased glioma grade and negatively related to γδ T cell infiltration. Although further examination should follow, we have concluded γδ T cells are the most HGG-reactive cells, and are suppressed by tumor hypoxia. If we used metformin to block tumor cell respiration, hypoxia-induced suppression of γδ T cells was reduced, which resulted in a recovery of their antitumor functions. Though IL-17A and IL-17F were not related to survival of HGG mice, NKG2D expression of IFN-γ-producing γδ T cells was critical for anti-HGG immunity. Due to high NKG2D-ligand expression of tumor cells, NKG2D-expressing γδ T cells were the most critical immune cells in the HGG microenvironment. In this study, anti-γδTCR antibody administration also abrogated γδ T cell-mediated antitumor functions. This finding suggested that γδTCR also participates in anti-HGG immunity. Despite lack of a direct connection, this study suggested dual ligation of γδTCR and NKG2D is needed, which could be the reason why other NKG2D-expressing cells, such as NK cells, did not respond to metformin treatment.

γδ T cells have been considered a good target for next-generation anti-brain tumor therapy (139). Among malignant brain tumors, GBM is the most frequent and aggressive tumor type (140). Despite traditional therapies, including surgery, radiotherapy, and chemotherapy, overall survival of GBM patients is around 1-2 years (141). Despite the recent development of immunotherapy, such as anti-PD-1 therapy, clinical trials of immunotherapy to treat GBM showed disappointed results (142). Although it is too early to definitively conclude, these negative results may be due to the poor immune profile of GBM microenvironment. GBM is classified as a “cold tumor,” which showing less neoantigen and immune cell infiltration compared to “hot tumors” (143). Thus, modulation of existing immune cells could have limitations. According to our results, γδ T cells could be a better alternative target for anti-GBM therapy (138). In addition, preferential infiltration of Vγ9Vδ2 T cells in the GBM patient tissues was also observed (26). Because pre-existing T cells are not sufficient to eradicate tumors, interest in adoptive cell therapy has gained traction (144). However, adoptive therapy using *in vitro* expanded conventional T cells has shown low effectiveness (145). It may be that expanded conventional T cells are derived from low mutational and neoantigen burden in combination with downregulated antigen processing which resulting in GBM immune evasion despite controversies (146–148). *In vitro* studies have shown γδ T cells have cytotoxicity against multiple GBM cells, but not normal brain cells (149). Vγ9Vδ2 T cells were also able to target glioma stem cells (GSCs). Stereotaxic administration of Vγ9Vδ2 T cells with TCR stimulation by bromohydrin pyrophosphate or zoledronate efficiently controlled GSC-derived brain tumors in animal models (150). However, splenocyte-derived γδ T cell injection did not increase survival period of immunocompetent GL261-bearing mice. Consistently, γδ T cell deficiency did not affect survival of mice (151). The authors of this study suggested that γδ T cells are highly apoptotic in the GBM microenvironment. Consistently, our group has proposed that tumor hypoxia may contribute to γδ T cell apoptosis in the GBM microenvironment (138). Thus, γδ T cell therapy combined with anti-hypoxia strategy could have a beneficial effect. Our study also showed γδ T cell therapy in combination with metformin or pretreatment of HIF1A inhibitor dramatically increased survival of tumor-bearing mice. In addition, chemotherapy-mediated cell death could be another detrimental factor for γδ T cell activity. Thus, engineered γδ T cells which are resistant to chemo/radiotherapy may be an alternative approach (152). Allogenic γδ T cell therapy has a distinct advantage because γδ T cells are not dependent on MHC-mediated antigen presentation. Thus, γδ T cell therapy for tumors, including GBM, is expected to be a “game changer”. Because the beneficial effect of γδ T cells in low-grade glioma (LGG) was clearer than HGG (138), γδ T cells may also have antitumor effects against other brain tumors, such as meningioma. Further studies should address the origins of γδ T cells (e.g. meninges, circulation), which ligands γδ T cells recognize, and mechanisms of γδ T cell infiltration (e.g. directly derived from peritumoral blood vessels, leptomeninges, choroid plexus).

## Conclusion

Several lines of evidence have demonstrated the contribution of γδ T cells to CNS inflammation, antitumor immunity, and maintenance of CNS homeostasis. Under homeostasis, IL-17A-producing γδ T cells are located in the meninges. IL-17A derived from γδ T cells regulates multiple brain functions, including memory formation and behaviors. Brain inflammation also induces parenchymal infiltration of multiple subsets of γδ T cells. Although it is difficult to completely understand due to the complexity of γδ T cell biology, it is clear that γδ T cells play a critical role in a number of brain diseases. Multiple studies have suggested IL-17A-producing γδ T cells are associated with inflammation initiation. On the other hand, IFN-γ-producing γδ T cells are beneficial for removing tumors and pathogens. Furthermore, γδ T cells tend to be associated with early onset of diseases rather than late stages. Thus, γδ T cells can be considered as an early sensor for inflammation and may act as a connecting bridge with further inflammation. Because γδ T cells actively surveil and rapidly respond to brain diseases, understanding their role is important for neuroimmunology research. Further study investigating different γδ T cell subsets in different contexts and at different time points will give critical insights into mechanisms regulating neuro-immune interactions.

## Author Contributions

JP, IK, and HL wrote the manuscript. All authors contributed to the article and approved the submitted version.

## Funding

This study was supported by the National Research Foundation of Korea (NRF-2021M3A9D3026428 and NRF-2021M3A9H3015688) funded by the Ministry of Science and ICT of Korea.

## Conflict of Interest

The authors declare that the research was conducted in the absence of any commercial or financial relationships that could be construed as a potential conflict of interest.

## Publisher’s Note

All claims expressed in this article are solely those of the authors and do not necessarily represent those of their affiliated organizations, or those of the publisher, the editors and the reviewers. Any product that may be evaluated in this article, or claim that may be made by its manufacturer, is not guaranteed or endorsed by the publisher.
